# A Shift From Non-operative Care to Surgical Fixation of Pediatric Humeral Shaft Fractures Even Though Their Severity Has Not Changed

**DOI:** 10.3389/fped.2020.580272

**Published:** 2020-11-05

**Authors:** Juuli Hannonen, Elina Sassi, Hanna Hyvönen, Juha-Jaakko Sinikumpu

**Affiliations:** ^1^Department of Children and Adolescents, Pediatric Surgery and Orthopaedics, Oulu University Hospital, Oulu, Finland; ^2^Research Unit for Pediatrics, Pediatric Neurology, Pediatric Surgery, Child Psychiatry, Dermatology, Clinical Genetics, Obstetrics and Gynecology, Otorhinolaryngology and Ophthalmology (PEDEGO Research Unit), University of Oulu, Oulu, Finland; ^3^Medical Research Center Oulu, Oulu, Finland

**Keywords:** humeral shaft fracture, children and adolescents, operative treatment, internal fixation, epidemiology, changing trends

## Abstract

**Introduction:** Humeral shaft fractures have traditionally been treated non-operatively due to their good union and low rate of functional impairment. In the recent years, upper extremity fractures and their operative treatment have increased in children. Nevertheless, the trends of humeral shaft fractures are not clear.

**Materials and Methods:** All children aged <16 years, with a humeral shaft fracture in the geographical catchment area of Northern Finland Hospital District, with a yearly child population-at-risk of ~86 000 from the year 2001 until the end of 2015 were included. There were 88 cases, who comprised the study population. Radiographs were available of all. Injury, patient, and treatment characteristics were reviewed from hospital databases.

**Results:** There was an increasing trend of surgical fixation of humeral shaft fractures during the 15 years' study period (β = 1.266, 95% CI 0.17 to 2.36, *p* = 0.035). However, we found no patient or fracture-related reasons that could have explained the increasing trend of surgical care. Comminuted fracture increased the risk of operative treatment 8-fold (Odds Ratio, OR 7.82, 95% CI 1.69 to 36.3, *p* = 0.009). Higher age, greater angular deformity or greater diameter of the humerus were not associated with the increased operation risk.

**Conclusions:** The treatment philosophy concerning pediatric humeral shaft fractures has presented a shift from conservative care to surgical fixation. To authors' understanding there is not evidence supporting the increasing rate of osteosynthesis.

## Introduction

Shaft fractures of the humerus comprise only 2–5% of all childhood fractures (1). They usually occur because of a direct trauma, where the trauma energy is usually high. They locate most often at the middle or distal third of the humerus (2). The radial nerve injury is the most usual primary complication of the humerus midshaft fracture, because of the close proximity of the nerve to the fracture site (3).

Due to forgiving physiology and great remodeling capacity of immature bone, most shaft fractures of the humerus in children have been treated by non-operative means. Functional arm bracing is feasible care, giving satisfactory support until stabilization (4) but hanging arm cast, coaptation splint or collar and cuff bandage are appreciated alternatives (5). Angular deformity up to 20–30° degrees has been suggested as acceptable in younger children (6), while 15–20° of angular deformation may be acceptable in older children (5). Actually, cosmesis may be greater sequelae than decreased function, because glenohumeral joint is a ball and socket joint with great movement ranges (7). Therefore, malunion usually doesn't decrease the function of upper extremity after humeral shaft fracture. Traditionally, indications for surgical fixation of humeral shaft have been multitrauma, open fracture and inability to reach and hold satisfactory reduction (2).

In adults, there have been several studies concerning the optimal fixation method, while plate/screws, elastic nails and rigid nails are all potential procedures (8). In children, the same procedures are available, including pinning, intramedullary rodding, screw fixation, compressive plating and external fixation (5). However, elastic stable intramedullary nailing (ESIN) has gained popularity since the literature in the field has increased recently (1, 7, 9, 10). ESIN is a straightforward technique and it can be performed both in antegrade and retrograde direction (7). The retrograde nailing is usually preferred because it doesn't damage the rotator cuff (2). The advantage of ESIN is also that the procedure is familiar to most pediatric orthopedists because it is widely used in other long-bone fractures in children. However, the published literature concerning the surgical techniques, in particular ESIN in pediatric humeral shaft fractures is scare and usually based on case reports, small series or expert opinions (2, 11).

Childhood upper extremity fractures have become more usual during the last decades (12, 13). Besides, there has been a changing trend in treatment of many childhood fractures, while the surgical fixation has increased instead of non-operative, orthopedic treatment e.g., in forearm shaft (14), elbow (15, 16), and proximal humerus (17, 18) fractures in children. The increasing rate of surgical treatment may be a result of higher demands of the patient and the family or a higher interest of the surgeons to progress in an operation room. However, there is a lack of research concerning the recent trends of treatment of the humeral shaft fractures in children which have been firmly treated non-operatively. The aim of this study was to evaluate the rate of surgical fixation and its potential trends and causes in children.

## Materials and Methods

### Study Design and Material

A population-based study consisted primarily of 99 children, 0–15 years of age, who had suffered from a humeral shaft fracture in the Northern Ostrobothnia Hospital District in Finland during the last 15 years until the year 2015. All cases who had the diagnose S42.3 in the International Classification of Diseases (ICD version 10), referring to the shaft fracture of humerus, at the area between proximal and distal metaphysis were first enrolled. However, non-habitants living outside of the study area, including two foreign children were excluded. The patients' original hospital charts and radiographs were reviewed in order to confirm the diagnosis and to determine the specific location and the type of the fracture, treatment type and results. Patients with a pathological fracture due to cysts (*N* = 6) or other bone disease were excluded: there were two cases with osteogenesis imperfecta and one with fibrous dysplasia. The final number of the patients was 88 and they formed the study population. Patient characteristics, radiographic findings and treatment particulars were analyzed by using hospital journals, operative charts and radiographs. The primary treatment was classified as operative vs. non-operative. Operative treatment comprised the procedures performed in an operation room under general anesthesia. Operatively treated cases were further classified based on the type of fixation (Table 1).

**Table 1:** The background characteristics of the study cases and the primary findings of the fractures.

		***N* (%)**
Age (Mean, SD^*^, Range)		9.4, 4.6, 0–15
Gender	Male	60 (68.2)
Side	Left	50 (56.8)
Trampoline injury		6 (6.8)
Downhill skiing		21 (23.9)
Motor vehicles		5 (5.7)
Other injury		56 (63.6)
Outdoor fracture		64 (73.6)
NAT^**^		0
Deformity with inspection		14 (15.9)
Tiggling nerve symptom		2 (2.3)
Compartment syndrome		0
Visible oedaema		18 (20.5)
Haematomae		6 (6.8)
Open fracture		1 (1.1)
Fracture location	Middle third	45 (51.1)
	Proximal third	19 (21.6)
	Distal third	24 (27.3)
Comminuted fracture		12 (13.6)
Operative treatment	All operated	18 (20.5)
	Plate and screws	8 (44.4)
	Intramedullary fixation	9 (50.0)
	External fixation	1 (5.6)
Opioid use	In-hospital use	31 (35.2)
	Out-hospital prescription	3 (3.4)
Follow-up visits > 4		9 (10.2)
Refracture		8 (9.1)

* *SD, Standard Deviation*;

** *NAT, Non-accidental trauma*.

The proportion (%) of the patients, who were treated surgically was determined. The potential trend in the surgical treatment over the entire study period was analyzed by using the linear regression analysis with 95% confidential intervals. The potential increasing or decreasing trend in the rate of surgical treatment was the main outcome. Analysis were performed in 3 years' periods, to reach satisfactory number of cases for each group (19).

The crude incidences of the fractures were reported for 100 000 children-at-risk. The population-at-risk was determined by using the official numbers by Statistics Finland. The number of child population was mean 85 697, and it changed between 83 842 and 88 093 during the study period. The frequencies and proportions of the categorical variables were reported. Mean, range and standard deviation were given for continuous variables. The risk (Odds ratio, OR, with its 95% Confidence intervals, CI) for operative treatment of humeral shaft fracture, according to the potential explanatory factors, was tested by binary logistic regression analysis. Male gender, higher age >9 years, bone thickness >30 mm, referring to more mature skeleton, great displacement without cortical bone contact, angular deformity >20° in any direction and comminuted fracture were taken as expected risk factors of operative treatment in this study. The differences of proportions were analyzed by using Standardized Normal Deviation (SND) test. The threshold of statistical difference was set at *P* <0.05 (5%) in all analyses, while all *P*-values were two-tailed. The data was analyzed by using IBM SPSS Statistics, version 24 (IBM SPSS Inc., Chicago, Illinois, United States) and StatsDirect Statistical Software Version 3.9 (StatsDirect Software, Inc., Ashwell, United Kingdom).

### Ethics Approval and Consent to Participate

The patients were not contacted for the study purpose and no Ethical Board evaluation was available according to official instructions by Ethical Committee of Northern Finland Hospital District, Oulu, Finland. However, an institutional approval was achieved in prior to study initiation. A consent to participate was not asked of any patient, because it was a registry-based study and the patients were not contacted.

## Results

### The Rate of Operative Treatment

**Figure 1:**
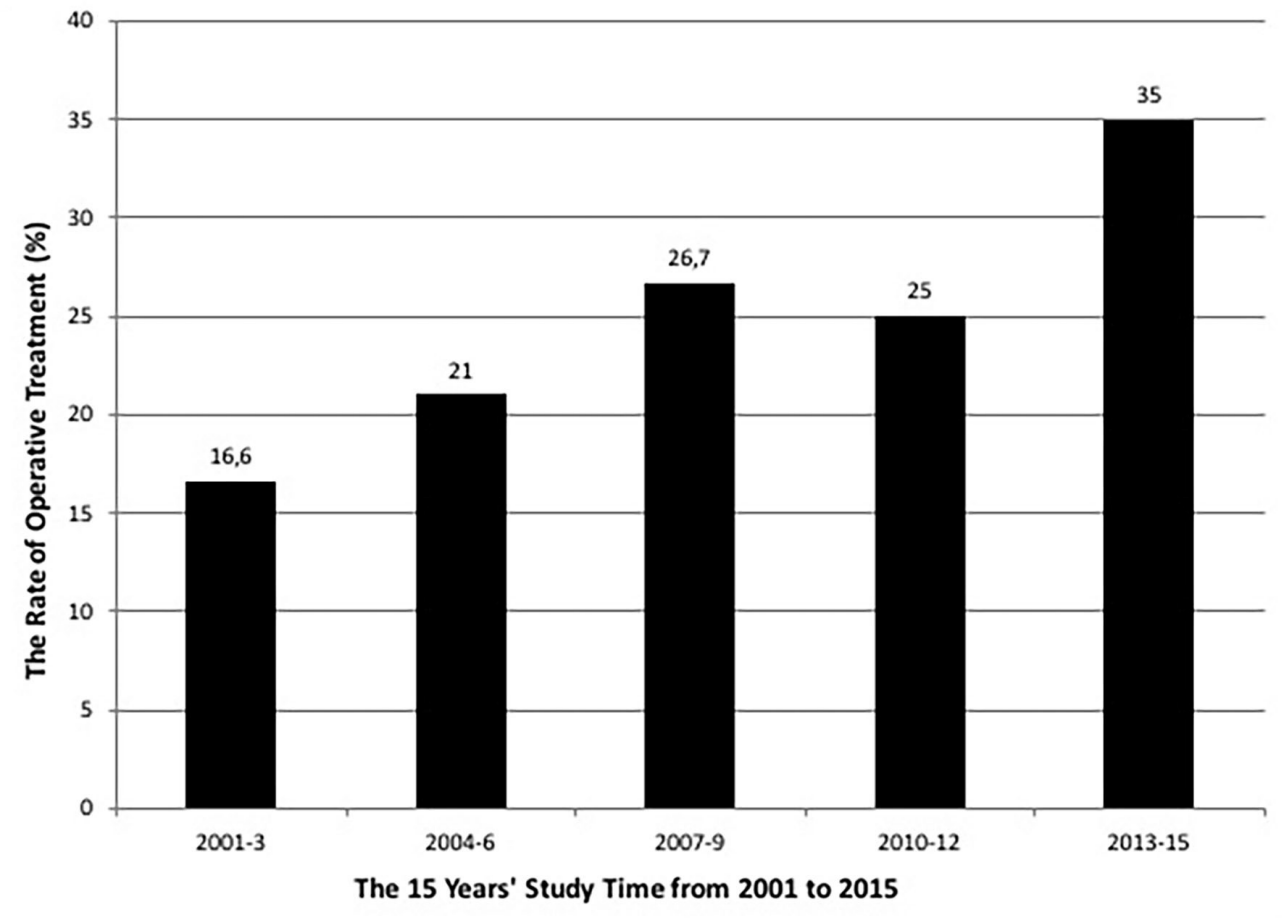
The rate of surgical treatment of the patients during the 15 years' study period from 2001 to 2015. The surgical treatment increased during the study time.

The rate of operative treatment of pediatric humeral shaft fractures was in total 20.5% (*N* = 18/88) during the fifteen years' study period. There was a steady increasing trend in the surgical fixation over the entire study time (β = 1.266 95% CI 0.17 to 2.36, *p* = *0.035*), as an alternative to the non-operative treatment (Figure 1).

However, there was no change in the potential explanatory factors for surgical treatment, such as greater fracture displacement or greater angulation (>20°) between the beginning and the end of the study period (Table 2). Neither was any change found in the characteristics of the patients from 2001–2003 to 2013–2015. Intramedullary nail or pin fixation (*N* = 9/18, 50.00%) and plate/screw fixation (*N* = 8/18, 44.44%) were usual surgical techniques while one (*N* = 1/18, 5.56%) was treated by external fixation.

**Table 2:** The background characteristics of the cases with humeral shaft fractures, by comparing the beginning of the study period to the end of the period.

		**2001–2003**	**2013–2015**	Diff.^*^	95% C.I.^**^	***p*-value**
		**N (%)**	**N (%)**	**(%)**	**(%)**	
Sex	Female	4 (23.5)	5 (33.3)			
	Male	13 (76.5)	10 (66.7)	9.8	−40.5 to 21.6	0.471
Age	>9 years	10 (58.8)	8 (53.3)	5.4	−28.0 to 38.1	>0.999
Location						
	Proximal third	6 (35.3)	4 (26.7)	8.6	−24.1 to 39.0	0.489
	Middle third	9 (52.9)	7 (46.6)	6.3	−27.6 to 38.8	>0.999
	Distal third	2 (11.8)	4 (26.7)	14.9	−43.3 to 13.6	0.229
>100% displacement		0 (0)	3 (20.0)	20.0	−45.6 to 0.93	0.053
Comminuted fracture		2 (11.8)	0 (0)	11.8	−10.4 to 34.8	0.243
>20° angular deformation		3 (17.6)	5 (33.3)	15.7	−45.2 to 15.1	0.265

*During the years 2001–2003 the total number of fractures were 17 and during the years 2013–2015 it was 15*.

*Tested by SND-test*,

* *Diff, Difference*;

** *Confidential interval*.

### Factors Affecting the Operative Treatment

Comminuted fracture, comprising more than two fractured bone parts, increased the risk of surgical fixation 8-fold (OR 7.82, 95% CI 1.69 to 36.27, *p* = 0.009) and the higher displacement of the fracture with lacking bone contact 7-fold (OR 6.91, 95% CI 1.21 to 39.56, *p* = 0.030), according to a binary logistic regression analysis. Higher age of the patient, greater dimensions of the skeleton (bone thickness > 30 mm), male gender or higher angular deformity (>20°) didn't associate with the increased risk of operative care (Table 3). One comminuted fracture with a large loose butterfly fragment was taken to be segmental; it was treated by using an external fixator.

**Table 3:** The risk for operative treatment, according to the potential explanatory risk factors, evaluated by using binary logistic regression analysis.

	OR^*^	95% C.I. ^**^		
		**Lower**	**Upper**	***p-value***
Male gender	0.671	0.157	2.857	0.589
Age >9 years	3.790	0.857	16.767	0.079
No bone contact	6.906	1.206	39.559	0.030
Angulation >20°	2.193	0.537	8.955	0.274
Bone thickness >30 mm	2.720	0.478	15.471	0.259
Comminuted fracture	7.823	1.687	36.272	0.009

* *OR, Odds Ratio*;

** *Confidential Interval*.

### Annual Incidence and Injury Characteristics

The annual incidence of humeral shaft fractures in children <16 years of age was mean 6.8/100 000 during the study period. The mean incidence was 4.17/100 000 in girls and 8.49/100 000 in boys (Diff. 4.3%, 95% CI −12.2 to 3.3%, *p* = 0.267).

There was no change in the annual incidence of the fractures during the study time; it was 6.74 / 100 000 in 2001–2003, compared with 5.69 / 100 000 in 2013–2015 (Diff. 1.1%, 95% CI −6.9 to 9.2%, *p* > *0.999*). Downhill skiing was the most usual recreational activity (*N* = 21, 23.9%), while trampolining was associated with only six (6.8%) fractures. Five cases (5.7%) associated with motor vehicles (Table 1).

## Discussion

The main finding of this research was that the surgical fixation of humeral shaft fractures presented an increasing trend during the 15 years' study period. The surgical stabilization has increased as an alternative to non-operative treatment, which is in line with our hypothesis. We analyzed also the potential explanatory factors of surgical interventions but found no significant change in patients' or fractures' characteristics during the study time that could have explained the changes. The severity of the fractures didn't change. Thus, as a conclusion, it seems clear that the increase in operatively treated cases during the study time has been nothing but a consequence of lower threshold for surgery during the end of the study period. This is an important finding, because the evidence supporting the increasing operative treatment is still sparse, to our understanding.

Humeral shaft fractures are usually expected to heal well, and surgical intervention has traditionally been reserved for complicated cases (2, 5). However, as an advantage, surgical stabilization permits early mobilization and return to normal daily activities. Further, over the time, a desire for more accurate alignment and shorter hospital stays have resulted in more frequent surgical care in children's fractures, in general (20). These same reasons may have affected to the remarkable increase of surgical treatment of humeral shaft fractures in the study area, too. Besides, required follow-up is much less frequent after surgical stabilization than non-operative care; radiographs should be taken weekly to exclude deformity worsening in non-operative care (5).

The increasing trend of treating humeral shaft fractures by operative means is significant but not surprising finding; there are previous reports of increasing operative treatment of many childhood fractures (15, 21, 22). Surgical treatment of femoral shaft fractures has increased (23) but in particular upper extremity fractures such as forearm fractures (24), supracondylar (12) and proximal humeral fractures (18) have been treated more usually with surgery. One explanation for the increased surgical treatment of long bone fractures as compared with non-operative treatment may be the increased popularity of elastic stable intramedullary nailing (ESIN). That sophisticated procedure has changed the way of thinking when treating pediatric diaphyseal fractures, including humeral shaft fractures (25). ESIN has given good rotational control and stable reduction with good Quick-DASH scores in humeral shaft fractures among children (26). Some experts use smooth metal pins for intramedullary fixation. However, plate/screw fixation is an important option for humeral shaft fractures at present, while comminuted fractures with loose fragments cannot be usually stabilized by ESIN (27). External fixation is required particularly in cases with large soft tissue injury (28, 29). However, we are aware that high-level literature concerning both the surgical treatment vs. conservative treatment of humeral shaft fractures and different surgical procedures has been sparse.

An important secondary finding of this study was that the incidence of humeral shaft fractures has held stable during the long study time of 15 years. This was an unexpected finding, in the light of current literature, while upper arm fractures generally increased by 39% between 1983 and 2005 in Helsinki, Finland (17). However, that study was performed in the completely urban capital area and its results are not straightforward generalizable to all living areas. Our finding of the stable incidence of humeral shaft fractures is different from the respective trend of forearm shaft fractures in the identical geographical study area: they increased 4-fold during a decade since 2000's (14). Nevertheless, while forearm fractures have become much more usual, hand and clavicle fractures have become less frequent (17). The reason for the different trends in the incidences of upper extremity fractures is unclear but we suggest that they are rather injury-related reasons than biological and anatomy-related reasons: trampolining e.g., has been the main reason for the increase of forearm shaft fractures (24) but only few isolated humeral shaft fractures were trampoline-related in this study. While forearm shaft fractures are usually a consequence of a fall against an out-stretched arm, humeral shaft fractures usually require higher trauma energy, such as a direct impact or a traffic accident (30). Humeral shaft fractures are usually not conventional fall injuries on the same level. In this study, the majority (73.6%) of the injuries occurred in outdoor activities, which fits well with the idea of higher trauma energy.

We found also that deformity, hematoma and edema were frequent findings with admission, but nerve complications were not usual. This is encouraging, keeping in mind that the radial nerve lays close to the humeral shaft and is vulnerable for damages. Only two cases with neuropraxia were found but they were temporary and resolved without any special intervention. Further, we found that the fractures usually located at the middle third shaft. This agrees with the previous literature and is reasonable, because the humeral shaft is narrow, the cortical bone is thinner and the external deforming forces in a case of injury are greater in this central area (30). In general, the closer the fractures are to the metabolically active growth plate, the better. We found that all fractures united and no ossification operation was needed, which fits well with the current knowledge of the good natural history (30, 31).

As strengths, this study was performed during the 15-years' study period, which is long enough time span for making conclusions about the epidemiological trends. All consecutive patients during the study time were preliminary enrolled to the study, and all their radiographs were reviewed to ensure the inclusion criteria. All radiographs were available. The patients living outside of the geographic catchment area were excluded. The patients who suffered from bone dysplasia and pathological fractures due to bone cyst (of any nature) were excluded. The inclusion was comprehensive, and the epidemiological conclusions were certain, and they are generalizable. The annual population-at-risk was available for both genders by the official national statistics. There are no other round-the-clock pediatric trauma units in the area and all potential cases were available to the study. However, if there have been some isolated cases who have been treated elsewhere, e.g., during their vacation or at the private clinics, the number must be small and would not have changed our main findings.

We recognize that the study is a subject to some criticism, too. Humeral shaft fractures are quite uncommon, comprising ~2–5% of all childhood fractures. Despite the appreciated study population with about 86.000 children, the number of fractures was still relatively small. Due to low annual frequencies, 3 nearest years were combined in the analyses to get sufficient number of cases in analyzing the potential trend of surgery. For the same reason, we didn't perform more subgroup analyses e.g., about the different osteosyntheses or different anatomical location of the fractures. Further, while the main finding of this study was the increasing trend of operative treatment, we were not able to make any closer evaluation about the different surgical methods and surgeons preferences in different conditions: because of the retrospective study design, no further information about the primarily injury were available and we were dependent on the information written in the journal charts. We recognized all the injury types to distinguish non-accidental trauma (NAT) but found no such case. All fractures were injury related. Further, as a limitation of this study, the long-term outcomes of the fractures were not available. We found that bone healing was complete in all cases, but we had not systematically collected information about function, subjective satisfaction or residual symptoms. Further studies are needed to evaluate the long-term functional recovery and the quality of life after humeral shaft fracture.

## Conclusion

The surgical treatment of pediatric humeral shaft fractures has increased in 15 years' study time, as an alternative to non-operative care, albeit there is no firm evidence supporting that. Level-1 studies concerning the treatment of this trauma are highly warranted. Contrary to many other pediatric upper extremity fractures, the overall incidence of humeral shaft fractures has not increased.

## Data Availability Statement

The raw data supporting the conclusions of this article will be made available by the authors, without undue reservation.

## Ethics Statement

The patients were not contacted for the study purpose and no Ethical Board approval was required, according to the official instructions by the Ethics Committee of Northern Finland Hospital District. Institutional approval was affirmed prior to study initiation from Oulu University Hospital.

## Author Contributions

JH had contributed in data collecting, analyzing, and writing. ES had contributed in writing and reviewing. HH had contributed in study design, data collecting, and reviewing. J-JS was initiative to study and contributed in planning, writing, and reviewing. All authors have contributed, read, and approved the manuscript.

## Conflict of Interest

J-JS was a member of European Paediatric Orthopaedic Society and European Paediatric Surgery Association, the assistant editor of Scandinavian Journal of Surgery, a member of editorial board of Journal of Children's Orthopaedics and a vice-chair of Finnish Paediatric Orthopaedic Society. He had received grants for scientific working by Foundation of Pediatric Research, Alma and K. A. Snellman foundation and Emil Aaltonen foundation. He had been a consulting surgeon for Bioretec ltd. The remaining authors declare that the research was conducted in the absence of any commercial or financial relationships that could be construed as a potential conflict of interest.
